# Kongestive Nephropathie – Ursachen, Diagnostik und Therapie

**DOI:** 10.1007/s00108-025-01894-5

**Published:** 2025-05-20

**Authors:** Manuel Wallbach, Stephan von Haehling, Michael Koziolek

**Affiliations:** 1 Klinik für Nephrologie und Rheumatologie, Universitätsmedizin Göttingen, Deutsches Zentrum für Herz-Kreislauf-Forschung, Robert-Koch-Straße 40, 37075 Göttingen, Deutschland; 2 Klinik für Kardiologie und Pneumologie, Universitätsmedizin Göttingen, Deutsches Zentrum für Herz-Kreislauf-Forschung, Robert-Koch-Straße 40, 37075 Göttingen, Deutschland

**Keywords:** Dopplersonographie, Renalvenöse Stase, Venöse Impedanz, „Venous excess ultrasound“, Renokardiales Syndrom, Ultrasonography, Doppler, Renal venous stasis, Venous impedance, Venous excess ultrasound, Reno-cardiac syndrome

## Abstract

Die kongestive Nephropathie (CN) ist eine Entität des kardiorenalen Syndroms, die wesentlich auf dem Boden einer venösen Kongestion und neurohormonellen Aktivierung entsteht. Eine Herzinsuffizienz, pulmonalarterielle Hypertonie, isolierte Trikuspidalklappeninsuffizienz und angeborene Herzfehler sind die häufigsten Ursachen. Es gibt bis dato keine allgemein akzeptierten diagnostischen Kriterien, jedoch scheint das Erfassen des intrarenalen venösen Blutflusses mittels Dopplersonographie die geeignetste Methode zu sein. Mit dieser Technik kann ein kontinuierlicher venöser Fluss (keine Kongestion) von den diskontinuierlichen Flussmustern pulsatil (leichte Kongestion), biphasisch (moderate Kongestion) und monophasisch (schwere Kongestion) differenziert werden. Der Venous Impedance Index und der Renal Venous Stasis Index sind zusätzliche dopplersonographische Kriterien zum Erfassen einer CN. Therapien mit Schleifendiuretika und/oder Natrium-Glukose-Kotransporter-2(SGLT-2)-Inhibitoren können eine venöse Kongestion nachweislich verbessern.

## Abstract (en)

Congestive nephropathy (CN) is an entity of the cardiorenal syndrome that essentially arises from venous congestion and neurohormonal activation. The most common underlying causes include heart failure, pulmonary arterial hypertension, isolated tricuspid valve insufficiency and congenital heart defects. Currently, there are no universally accepted diagnostic criteria; however, the most suitable method appears to be the recording of intrarenal venous blood flow using Doppler sonography. A distinction can be made between continuous venous flow (no congestion) and discontinuous flow patterns, categorized as pulsatile (mild), biphasic (moderate) and monophasic (severe congestion). The venous impedance index (VII) and the renal venous stasis index (RVSI) are additional Doppler sonographic criteria for detecting CN. Evidence supports the efficacy of loop diuretics and/or the administration of sodium-glucose cotransporter 2 (SGLT2) inhibitors in the management of venous congestion.

## Lernziele

Nach Lektüre dieses Beitrags

- können Sie die häufigsten Ursachen einer kongestiven Nephropathie benennen.
- interpretieren Sie einen Kreatininanstieg bei Patienten mit Herzinsuffizienz zuverlässig.
- können Sie die differenzialdiagnostischen Schritte im Falle eines Kreatininanstiegs bei Patienten mit Herzinsuffizienz sicher einleiten.
- können Sie die venösen Flussprofile einer kongestiven Nephropathie unterscheiden.

## Einleitung

Ein breites Spektrum **klinischer Begleiterkrankungen**

Klinische Begleiterkrankungen

spielt bei Patienten mit **Herzinsuffizienz**

Herzinsuffizienz

(HI) eine signifikante Rolle im Zusammenhang mit dem Auftreten ungünstiger Ereignisse und einem erhöhten Sterberisiko. Unter den Begleiterkrankungen hat sich die **Nierenfunktionsstörung**

Nierenfunktionsstörung

als hochprävalente **prognostische Variable**

Prognostische Variable

erwiesen. Fast 60 % der Patienten, die wegen akut dekompensierter HI ins Krankenhaus eingeliefert werden, sind von ihr betroffen. Der Einfluss dieser Störung auf die Prognose, die Dauer des Krankenhausaufenthalts und den Bedarf an Intensivpflege nimmt mit zunehmendem Grad der Niereninsuffizienz bzw. -schädigung zu [1]. Eine große Metaanalyse mit über 1.000.000 HI-Patienten ergab, dass das Vorhandensein einer **chronischen Nierenerkrankung**

Chronische Nierenerkrankung

(„chronic kidney disease“ [CKD]) das allgemeine Sterberisiko verdoppelt [2].

HI-bedingte Nierenfunktionsstörungen äußern sich hauptsächlich in Schwankungen der errechneten glomerulären Filtrationsrate (**eGFR**

eGFR)

aufgrund von hämodynamischen und neurohormonellen Faktoren, die die Leistung einzelner Nephrone durch eine Verringerung des renalen Blutflusses (RBF) negativ beeinflussen [3]. Dabei hat eine Erhöhung des **zentralvenösen Drucks**

Zentralvenöser Druck

einen höheren Einfluss auf das Auftreten einer sich verschlechternden Nierenfunktion als ein eingeschränkter Herzindex [4]. Dies hat zum Begriff der **kongestiven Nephropathie (CN)**

kongestive Nephropathie (CN)

geführt. Umgekehrt können eine akute oder chronische Nierenschädigung und die damit einhergehenden Folgen die kardiale Funktion negativ beeinflussen (**renokardiales Syndrom**

Renokardiales Syndrom).

## Fallbeispiel

**Abb. 1:**
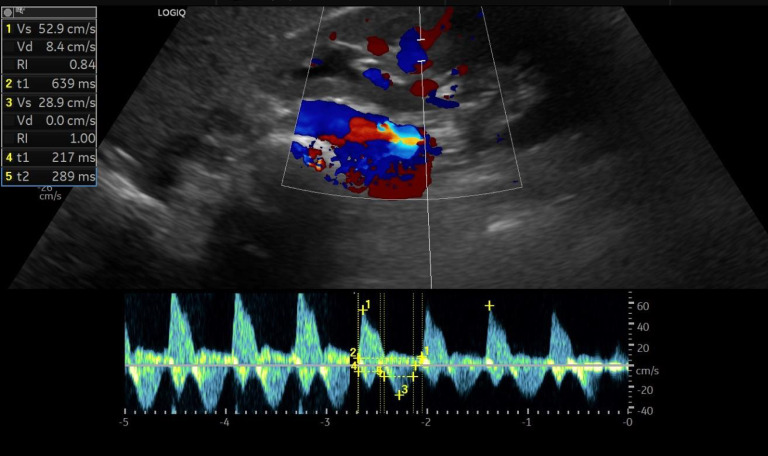
Intrarenale Dopplersonographie der rechten Niere. (Quelle: eigene Abbildung). In der Duplexsonographie Ableitung der A. interlobaris (oberhalb der Nulllinie) und V. interlobaris (unterhalb der Nulllinie). Relevante Messgrößen: IRVF-Muster biphasisch, VII = 1,0 und RVSI = 0,21. *IRVF* intrarenaler venöser Fluss, *RVSI* Renal Venous Stasis Index (Index der renalvenösen Stase), *VII* Venous Impedance Index (Index der venösen Impedanz)

Ein 65 Jahre alter Patient wird von seiner Hausärztin zur stationären Behandlung eingewiesen. Bei ihm sind ein metabolisches Syndrom, eine ischämische Kardiomyopathie mit einer linksventrikulären Ejektionsfraktion (LVEF) von 30 % sowie eine chronische Nierenerkrankung im CKD-Stadium G3a A3 bekannt. Er wurde bislang mit Ramipril 5 mg 1‑0‑1, Metoprolol retard 47,5 mg 1‑0‑1, Spironolacton 25 mg 1‑0‑0, Torasemid 5 mg 1‑0‑0 und Metformin 500 mg 1‑0‑1 behandelt. Aufgrund der häufigen Neigung zur Hyperkaliämie unter Angiotensin-converting-enzyme(ACE)-Hemmer/Spironolacton wurde zuletzt die Dosierung beider Arzneistoffe reduziert (Ramipril 5 mg ½‑0-½ und Spironolacton 25 mg jeden zweiten Tag). Darunter kam es jedoch zur Verschlechterung des kardialen Zustands. Der Patient berichtet bei Aufnahme über eine Dyspnoe bei geringer Belastung (New-York-Heart-Association[NYHA]-Stadium III) und hat deutliche Unterschenkelödeme. Auskultatorisch hört man Rasselgeräusche über allen Lungenfeldern. In der Röntgenuntersuchung des Thorax sehen Sie eine beidseitige pulmonale Stauung, Infiltrationen sind nicht auszuschließen. Sie erhalten die Ergebnisse der Aufnahmelaboruntersuchung (Tab. 1) und führen daraufhin eine Sonographie und Dopplersonographie der rechten Niere durch (Abb. 1).

**Tab. 1:** Relevante Laborparameter bei Aufnahme

Parameter	Normalbereich	Patient
Hämoglobin (g/l)	12,0–16,0	11,0
pH	7,35–7,45	7,221
Basenüberschuss (mmol/l)	−2–3	−11,1
Standardbicarbonat (mmol/l)	21–27	14,8
C‑reaktives Protein (mg/dl)	0,1–0,5	67,1
Glukose aus BGA (mg/dl)	70–110	261
Natrium-BGA (mmol/l)	135–148	121
Kalium (mmol/l)	3,5–5,0	5,9
Kreatinin (mg/dl)	0,5–1,2	2,7
eGFR nach CKD-EPI (ml/min pro 1,73 m^2^)	> 90	28
Harnstoff (mg/dl)	< 71	163
Albumin im Spontanurin, UACR (mg/g Kreatinin)	< 30	972
NT-proBNP (ng/l)	< 125	20.387

*BGA* Blutgasanalyse, *CKD-EPI* Chronic Kidney Disease Epidemiology Collaboration, *eGFR* errechnete glomeruläre Filtrationsrate, *NT-proBNP* N‑terminales natriuretisches Propeptid vom B‑Typ, *UACR* Urin-Albumin-Kreatinin-Quotient

## Definition und Ursachen

Der Begriff CN wurde eingeführt, um eine klinische Entität der Nierenfunktionsstörung zu beschreiben, die mit **venöser Stauung**

Venöse Stauung

und **verminderter Nierenperfusion**

Verminderte Nierenperfusion

einhergeht und vornehmlich bei Patienten mit **kardiorenalem Syndrom**

Kardiorenales Syndrom

beobachtet wird. Bis zum heutigen Tag gibt es für diesen hämodynamischen Phänotyp der Nierenfunktionsstörung keine einheitlich akzeptierte Definition [5]. Ein hoher zentraler Venendruck bedingt eine komplexe Pathogenese aus hormonellen Veränderungen und endothelialer Aktivierung, Leberdysfunktion, Entwicklung von Aszites, erhöhtem intraabdominellem Druck („intraabdominal pressure“ [IAP]), Ischämie der Darmschleimhaut (mit Übertritt bakterieller Toxine), Entzündung, oxidativem Stress und gesteigerter tubulärer Natriumrückresorption in den Nieren mit konsekutiver Volumenüberlastung, was wiederum zu einer weiteren Belastung des rechten Ventrikels führt [6].

Die HI ist wahrscheinlich die häufigste Erkrankung, die mit einer CN einhergeht; weitere Erkrankungen sind eine **pulmonalarterielle Hypertonie**

Pulmonalarterielle Hypertonie

(PAH) jeglicher Ursache, die **isolierte Trikuspidalinsuffizienz**

Isolierte Trikuspidalinsuffizienz

und **angeborene Herzfehler**

Angeborene Herzfehler.

Eine CN sollte auch bei Patienten vermutet werden, bei denen sich die Nierenfunktion nach **Entwässerung**

Entwässerung

durch Diuretika, HI- und PAH-spezifische Therapie oder mechanische Maßnahmen (beispielsweise Aszitesparazentese) verbessert [6].

### Merke.

HI, pulmonalarterielle Hypertonie, isolierte Trikuspidalklappeninsuffizienz und angeborene Herzfehler sind die häufigsten Ursachen der CN.

## Pathophysiologie

Die Pathophysiologie der CN ist komplex und beinhaltet im Wesentlichen zwei Mechanismen:

- Venöse Stauung
- Neurohormonelle Aktivierung

### Venöse Stauung

Der mittlere arterielle Druck („mean arterial pressure“ [**MAP**

MAP])

stellt nicht die einzige Determinante der Nierendurchblutung dar. Der RBF wird durch den **Druckgradienten**

Druckgradient

zwischen Zufluss- und Abflussdruck der Nieren sowie durch den **vaskulären Strömungswiderstand**

Vaskulärer Strömungswiderstand

determiniert [7]. Der **Zuflussdruck**

Zuflussdruck

der Niere steht in engem Zusammenhang mit dem MAP, während der **Abflussdruck**

Abflussdruck

durch den **renalen Venendruck**

Renaler Venendruck

oder den IAP bestimmt wird [7]. Es besteht eine kurvenförmige (in Form eines „umgekehrten U“) Beziehung zwischen zentralem Venendruck und glomerulärer Filtrationsrate (GFR; [8]). Ein erhöhter renaler Venendruck (während der Hyperhydratation) kann zunächst zu einem Anstieg der GFR führen, indem er eine glomeruläre Hyperfiltration induziert, solange der Druck im proximalen Tubulus unter dem glomerulären Netto-Ultrafiltrationsdruck (normalerweise ~20 mm Hg) bleibt [6].

Der normale Blutfluss in den zentralen Venen, einschließlich der Lebervenen, ist pulsatil. Diese **Pulsatilität**

Pulsatilität

spiegelt die normalen Druckschwankungen im rechten Vorhof wider, die während des normalen Herzzyklus auftreten. Wenn keine Stauung vorliegt, führt das **dehnbare Lumen**

Dehnbares Lumen

der peripheren Venen zu einer Abschwächung dieses pulsatilen Musters in den weiter distal gelegenen Venen und Venolen. Daher sind die normalen Flussmuster in der Pfortader und den Nierenvenen kontinuierlich oder nur leicht pulsierend [7]. Bei venöser Stauung sind die maximal gedehnten Venenwände nicht in der Lage, den retrograden Fluss aufzunehmen und die Druckübertragung auf die distalen Venolen und Kapillarbetten zu verhindern [9]. Die Erhöhung des zentralen Venendrucks wird direkt auf die Nierenvenen übertragen, da der venöse Gefäßwiderstand vernachlässigbar ist [6]. Da die **eingekapselte Niere**

Eingekapselte Niere

nur wenig Raum zur Ausdehnung hat, steigt der interstitielle hydrostatische Druck der Niere. Dieser Anstieg kann anfänglich durch eine kompensatorische Zunahme des renalen Lymphflusses kompensiert werden, jedoch resultiert eine anhaltende Erhöhung des zentralvenösen Drucks in einer Reduktion des venösen und lymphatischen Abflusses sowie im Spätstadium in einer Reduktion des arteriellen Zuflusses. Das postglomeruläre vaskuläre und tubuläre Netzwerk ist ein **Niederdrucksystem**

Niederdrucksystem,

sodass Erhöhungen des interstitiellen Nierendrucks zu einer Kompression oder einem Verschluss von Gefäßen und Tubuli führen [6]. Da die GFR direkt proportional mit dem RBF und umgekehrt proportional mit dem interstitiellen Druck verknüpft ist, sinkt sie bei Zunahme des renalen Venendrucks [1]. Bei einer venösen Stauung wird der unter physiologischen Bedingungen kontinuierliche intrarenale venöse Fluss (**IRVF**

IRVF)

unterbrochen, wobei die Unterbrechungen mit zunehmender Stauung länger werden.

### Neurohormonelle Aktivierung

Durch den reduzierten RBF sinkt die Filtrationsfraktion der Niere und es gelangt damit weniger Natrium und auch Chlorid in den Primärharn. Dies führt an der **Macula densa**

Macula densa

zu einer **Reninfreisetzung**

Reninfreisetzung

aus dem juxtaglomerulären Apparat, was eine **maladaptive Stimulation**

Maladaptive Stimulation

des Renin-Angiotensin-Aldosteron-Systems (RAAS) mit einer verstärkten Natriumretention stromabwärts bedingt [3]. Renin wiederum induziert eine Dilatation der glomerulären afferenten Arteriole [2]. Die neuronalen Verbindungen zwischen Geweben und Organen, insbesondere das sympathische Nervensystem, sind sowohl bei HI als auch bei Niereninsuffizienz überaktiviert, wogegen der Parasympathikotonus herabgesetzt ist [10]. Im **Splanchnikusbereich**

Splanchnikusbereich

sind bis zu 50 % des gesamten Blutvolumens in den Venen lokalisiert. Als Folge der **Sympathikusüberaktivität**

Sympathikusüberaktivität

bei HI kommt es zu einer **Vasokonstriktion**

Vasokonstriktion,

durch die ein Teil des Blutvolumens aus dem Splanchnikusbereich in das zirkulierende Kompartiment verschoben wird. Dies verstärkt die renale, intestinale und mesenteriale venöse Stauung [6]. Eine solche Verschiebung wird auch durch die Begriffe „ungestresstes Volumen“ (Blut im splanchnischen Gefäßkompartiment) und „gestresstes Volumen“ (Blut, das sich hauptsächlich im arteriellen System und in den nichtsplanchnischen Venen befindet) beschrieben. „Gestresstes“ Volumen trägt zur **kardialen Belastung**

Kardiale Belastung

bei und verstärkt eine Dekompensation [11].

#### Merke.

Die venöse Stauung und die neurohormonelle Aktivierung kennzeichnen die Pathogenese der CN.

## Diagnostische Kriterien der CN

Die Diagnostik der CN besteht in einer Kombination aus klinischen, laborchemischen und bildgebenden Verfahren.

### Klinik.

Klinisch präsentieren sich die Patienten meist mit Symptomen der auslösenden Erkrankung, in der Regel in Zusammenhang mit variablen **Überwässerungszeichen**

Überwässerungszeichen

(Beinödeme, Anasarka, Aszites, Pleuraerguss, gestaute Jugularvenen; [12]).

### Laboruntersuchung.

Eine Vielzahl gemessener Mediatoren und Marker ist bei einer CN verändert [11]. Bis dato existieren aber keine etablierten spezifischen Laborparameter zur Detektion der CN. Erhöhte Werte **natriuretischer Peptide**

Natriuretische Peptide

fungieren als Indikatoren für eine kardiale und intravaskuläre Stauung. Zur Beurteilung der Nierenfunktionsstörung werden im Wesentlichen die bekannten Biomarker Kreatinin, eGFR, Harnstoff(‑Stickstoff) und Cystatin C verwendet [5]. Zum Erfassen häufig auftretender Begleitprobleme wie Hyponatriämie, Hyperkaliämie und metabolischer Azidose sollte eine **venöse Blutgasanalyse**

Venöse Blutgasanalyse

erfolgen. Eine **Albuminurie**

Albuminurie

findet sich häufig bei HI, auch ohne typische Begleiterkrankungen wie Diabetes mellitus, Bluthochdruck oder CKD [11]. Im Rahmen einer Kongestion kann es zu einem Anstieg der Albuminurie kommen [6], die bei Dekongestion wieder rückläufig ist [13]. Patienten mit HI zeigen oft eine eingeschränkte **Natriurese**

Natriurese,

die im Zusammenhang mit einer Dekompensation weiter rückläufig ist. Die Natriurese wiederum ist invers mit der 24 h-Diurese assoziiert [11].

Die American Heart Association (AHA) schlägt zur besseren Charakterisierung der Nierenfunktionsstörung bei fortgeschrittener HI vor zu prüfen, ob eine **reversible Nierenfunktionsstörung**

Reversible Nierenfunktionsstörung

vorliegt. Im Grundansatz geht man davon aus, dass die GFR das Produkt aus der Anzahl funktionsfähiger Nephrone multipliziert mit der Leistung (GFR) eines einzelnen Nephrons ist. Eine Reduktion der **Nephronenzahl**

Nephronenzahl

charakterisiert eine **irreversible Nierenfunktionsstörung**

Irreversible Nierenfunktionsstörung,

wohingegen die **Einzelnephron-GFR**

Einzelnephron-GFR

von reversiblen Faktoren wie dem Ultrafiltrationskoeffizienten, den hydrostatischen Drücken des Bowman-Kapsel-Raums und der glomerulären Kapillare sowie von deren onkotischen Drücken abhängt. Faktoren, die für eine Irreversibilität sprechen, sind das Ausbleiben einer Besserung der GFR trotz Optimierung des rechtsatrialen Drucks, des Herzindex oder des MAP sowie auch klassische Begleiterkrankungen und -phänomene der chronischen Niereninsuffizienz, wie das Vorhandensein einer (höhergradigen) Albuminurie, eines sekundären Hyperparathyreoidismus oder einer renalen Anämie und auch sonographische Zeichen der chronischen Niereninsuffizienz [3].

### Bildgebung.

Bei venöser Stauung sind die maximal gedehnten **Venenwände**

Venenwände

nicht in der Lage, den **retrograden Fluss**

Retrograder Fluss

aufzunehmen und damit die Druckübertragung auf die distalen Venolen und Kapillarbetten zu kompensieren. Der Blutfluss in den zentralen Venen (untere Hohlvene und Lebervenen) ist von Natur aus pulsierend; diese Pulsatilität hängt mit den Druckänderungen zusammen, die bei jedem **Herzzyklus**

Herzzyklus

auftreten. Im Gegensatz dazu ist der normale Blutfluss in den kleinen Venolen und Kapillarbetten der Bauchorgane minimal pulsierend [9]. Zu den Ultraschallmethoden, die zur Beurteilung der venösen Stauung eingesetzt werden, gehören die Messung des Durchmessers der V. cava inferior (**VCI**

VCI)

sowie die Bestimmung ihres **Kollapsindex**

Kollapsindex

[9]. Ein Durchmesser der VCI von < 21 mm, wobei diese atemabhängig um mindestens 50 % kollabiert, zeigt einen normalen rechtsatrialen Druck an [14].

Die **intrarenale Dopplersonographie**

Intrarenale Dopplersonographie

(IRD) kann den **renalvenösen Fluss**

**Abb. 2:**
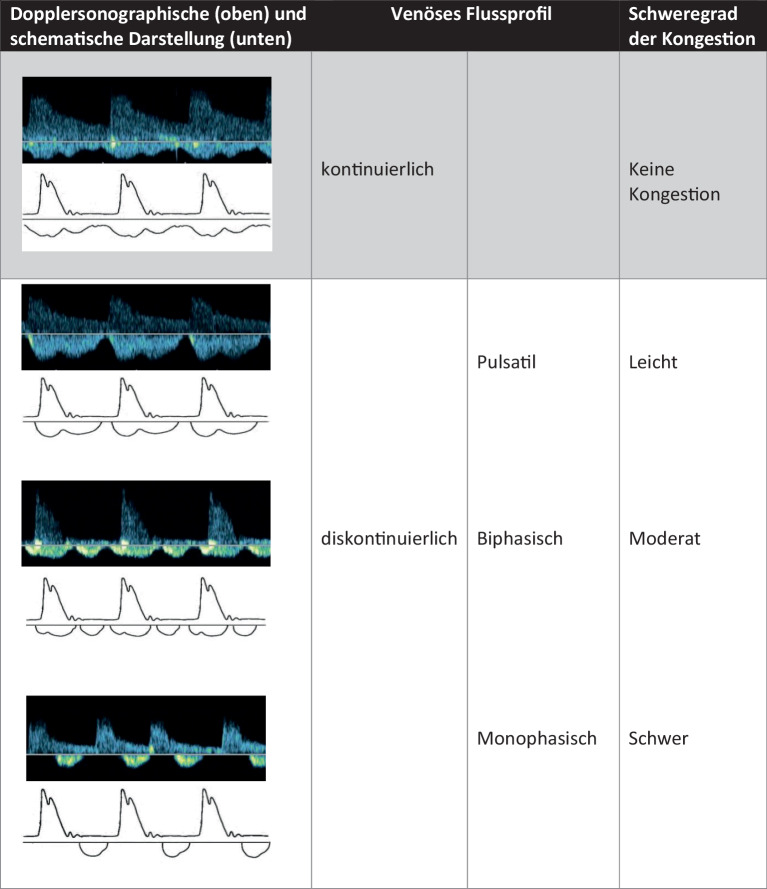
Stadien der venösen Kongestion. Oberhalb der Nulllinie wird der arterielle, unterhalb der Nulllinie der venöse Fluss dargestellt. (Modifiziert nach [57])

Renalvenöser Fluss

quantifizieren und hilft bei der Beurteilung der Kongestion. Der normale IRVF in den Venen ist kontinuierlich (Abb. 2). Da neben der **Druckübertragung**

Druckübertragung

des erhöhten rechtsatrialen Drucks auch die Auswirkungen eines **interstitiellen Ödems**

Interstitielles Ödem

erfasst werden sollen, erfolgt die IRD im Bereich der **Interlobarvenen**

Interlobarvenen.

Bei einer venösen Kongestion wird der IRVF unterbrochen, wobei die Unterbrechungen mit zunehmender Kongestion länger werden. Die rechte Niere wird in der langen und kurzen Achse von der mittleren Axillarebene in Linksseitenlage etwa auf Höhe des 10. Interkostalraums mit einem konvexen oder Sektorschallkopf (2,5–5 MHz) dargestellt. Die **Strömungsskala**

Strömungsskala

(Pulsrepetitionsfrequenz) sollte individuell auf eher niedrige Strömungsgeschwindigkeiten (vorzugsweise um 20 cm/s) eingestellt sein. Die Einstellung der Skala sollte dahingehend optimiert werden, dass die Amplitude des Signals maximiert wird [14]. Druckerhöhungen innerhalb des **intrakapsulären Raums**

Intrakapsulärer Raum

führen zum Kollabieren der Nierenvenen, was einen diskontinuierlichen IRVF bedingt [14]. Es konnte festgestellt werden, dass ein Anstieg des **zentralen Venendrucks**

Zentraler Venendruck

zu einer Zunahme der **Amplitudenvariation**

Amplitudenvariation

führt, wobei sich die minimale Geschwindigkeit sukzessive dem Nullpunkt annähert und schließlich zu einem vorzeitigen diskontinuierlichen oder pulsatilen Fluss führt. Bei weiterer Zunahme konnte ein **biphasisches Muster**

Biphasisches Muster

mit zwei getrennten Flussphasen während eines Herzzyklus identifiziert werden, und in schwerwiegenden Fällen kann der IRVF ein **monophasisches Muster**

Monophasisches Muster

aufweisen, mit einer einzigen Flussphase in der Diastole (Abb. 2). Die Analyse des IRVF-Musters gestattet somit eine **semiquantitative Bewertung**

Semiquantitative Bewertung

der Auswirkungen des zentralen Venendrucks auf die Hämodynamik der Nieren [14].

Andere quantitative Messgrößen sind der Index der venösen Impedanz (Venous Impedance Index [**VII**

VII])

 und der Index der renalvenösen Stase (Renal Venous Stasis Index [**RVSI**

RVSI]).

**Abb. 3:**
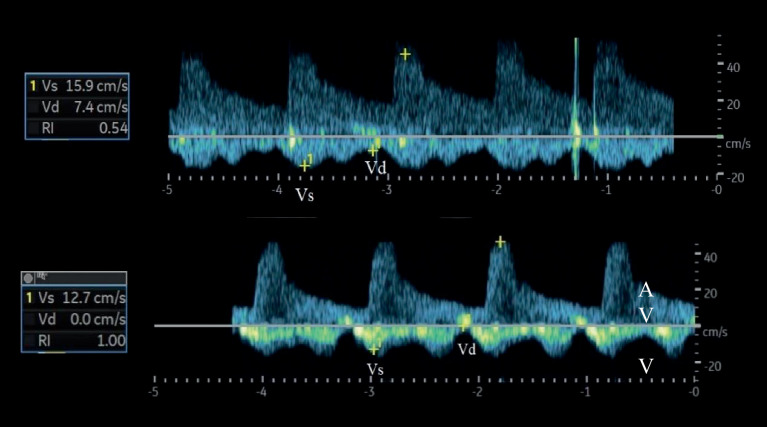
Berechnung des VII (in der Abbildung als RI bezeichnet) mithilfe der venösen Flusskurve bei einem kontinuierlichen (*oben*) und einem pulsatilen Flussprofil (*unten*). VII = (Vs−Vd)/Vs. *A* arterieller Fluss, *V* venöser Fluss, *Vd* enddiastolische Flussgeschwindigkeit, *VII* Venous Impedance Index (Index der venösen Impedanz), *Vs* maximale systolische Flussgeschwindigkeit. (Modifiziert nach [57])

**Abb. 4:**
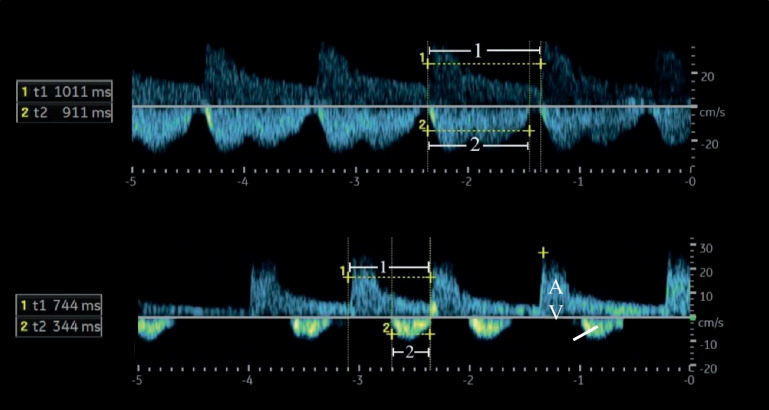
Berechnung des RVSI mithilfe der arteriellen und venösen Flusskurven bei einem pulsatilen (*oben*) und einem monophasischen Flussprofil (*unten*). 1: Dauer des Herzzyklus; 2: Dauer des venösen Flusses. Bei der pulsatilen Flusskurve beträgt der RVSI (1011−911)/1011 = 0,09. Bei der monophasischen Flusskurve beträgt der RVSI (744−344)/744 = 0,5. *A* arterieller Fluss, *RVSI* Renal Venous Stasis Index (Index der renalvenösen Stase), *V* venöser Fluss. (Modifiziert nach [57])

Der VII berechnet sich nach folgender Formel: VII = (maximale systolische Flussgeschwindigkeit Vs − enddiastolische Flussgeschwindigkeit Vd)/maximale systolische Flussgeschwindigkeit Vs. Bei einem kontinuierlichen Flussprofil nimmt der VII einen Wert < 1 an; mit zunehmendem Grad der Kongestion nähert er sich dem Wert 1 an (Abb. 3). Der RVSI gibt den Anteil des Herzzyklus an, in dem kein renalvenöser Abfluss vorhanden ist (Abb. 4). Der RVSI berechnet sich nach folgender Formel: RVSI = (Herzzykluszeit − venöse Flusszeit)/Herzzykluszeit. Er kann einen Wert von 0 bis 1 annehmen, wobei ein höherer Wert einen höheren Grad der Kongestion anzeigt. Ein hoher RVSI korreliert mit dem rechtsatrialen Druck, dem Durchmesser der unteren Hohlvene sowie der Rate kardialer Ereignisse [15]. Das Erfassen kongestiver (doppler-)sonographischer Veränderungen in den abdominellen Gefäßen (VCI, Pfortader, Lebervenen, Nierenvenen) wurde auch unter dem Begriff VEnous excess UltraSound (**VExUS**

VExUS)

zusammengefasst [16]. Es sollte berücksichtigt werden, dass Patienten mit HI, selbst im scheinbar stabilen Zustand, häufig (30–40 %) dopplersonographische Zeichen einer renalvenösen Kongestion aufweisen können [17]. Zudem zeigen bis zu 40 % der Patienten, die wegen akuter dekompensierter HI aufgenommen wurden, klinische Anzeichen einer **residualen Kongestion**

Residuale Kongestion

zum Zeitpunkt der Entlassung, was mit einem höheren Risiko für Mortalität und Wiederaufnahme verbunden ist [18].

Bei der Analyse der Parameter der renalvenösen Kongestion ist zu berücksichtigen, dass pathologische Veränderungen dieser Parameter nicht ausschließlich durch einen venösen Rückstau vor dem rechten Herzen verursacht werden. Sie können auch durch andere Faktoren beeinflusst werden, beispielsweise durch einen erhöhten IAP, eine obstruktive Nephropathie oder eine Nierenschwellung im Rahmen einer akuten Nierenschädigung.

### Histologische Veränderungen der CN.

Histopathologische Untersuchungen bei Menschen mit isolierter CN sind eine Seltenheit, da eine verminderte eGFR in der Regel auf eine Nephrosklerose und diabetische Nephropathie zurückgeführt wird [6]. Im Fallbericht einer akuten venösen Kongestion wurden normale Glomeruli und eine überwiegend normale tubuläre Zelldifferenzierung beobachtet. Neben fokal akzentuierten, geringgradigen akuten tubulären Schäden und kleinen Bereichen interstitieller Fibrose und tubulärer Atrophie wurden vor allem deutlich erweiterte peritubuläre Venen und Kapillaren festgestellt. Die Rolle einer lang anhaltenden Kongestion bei der Nierenfibrose ist jedoch nach wie vor Gegenstand kontroverser Diskussionen [19]. Somit ist die histologische Diagnostik (bis dato) nicht zielführend, es sei denn zum Ausschluss ggf. individuell vorliegender Differenzialdiagnosen.

### Merke.

Es gibt derzeit keine validierten Diagnosekriterien für eine CN. Zu den typischen Veränderungen zählen klinische Kongestionszeichen, eine Erhöhung des N‑terminalen natriuretischen Propeptids vom B‑Typ (NT-proBNP), Anstieg des Serumkreatinins und Albuminurie sowie dopplersonographische Kongestionskriterien (diskontinuierliches IRVF-Muster, VII-Erhöhung, RVSI-Abfall).

## Fallbeispiel (Fortsetzung)

Die durchgeführte laborchemische und apparative Diagnostik zeigt einen relevanten Abfall der eGFR von 45 auf aktuell 28 ml/min pro 1,73 m^2^, eine metabolische Azidose, eine Hyperkaliämie, eine Infektkonstellation sowie Zeichen einer akuten kardialen Dekompensation. Die IRD zeigt ein biphasisches Flussprofil, der VII liegt bei 1,0 und der RVSI bei 0,21, entsprechend einer moderaten venösen Kongestion.

## Therapie

Die Therapieziele bei Patienten mit CN umfassen eine vollständige und effektive **Entstauung**

Entstauung

und **Entwässerung**

Entwässerung

zur Vermeidung von Restvolumenüberladung, die Sicherstellung eines **angemessenen Perfusionsdrucks**

Angemessener Perfusionsdruck

zur Aufrechterhaltung der Organperfusion sowie die konsequente Fortführung einer leitliniengerechten medikamentösen Therapie [20].

### Entstauungs‑/Entwässerungstherapie

#### Angepasste Diuretikadosierungen

Die Leitlinien der European Society of Cardiology (ESC) empfehlen bei akuter kardialer Dekompensation mit Nachweis einer Kongestion den Einsatz von **Schleifendiuretika**

**Abb. 5:**
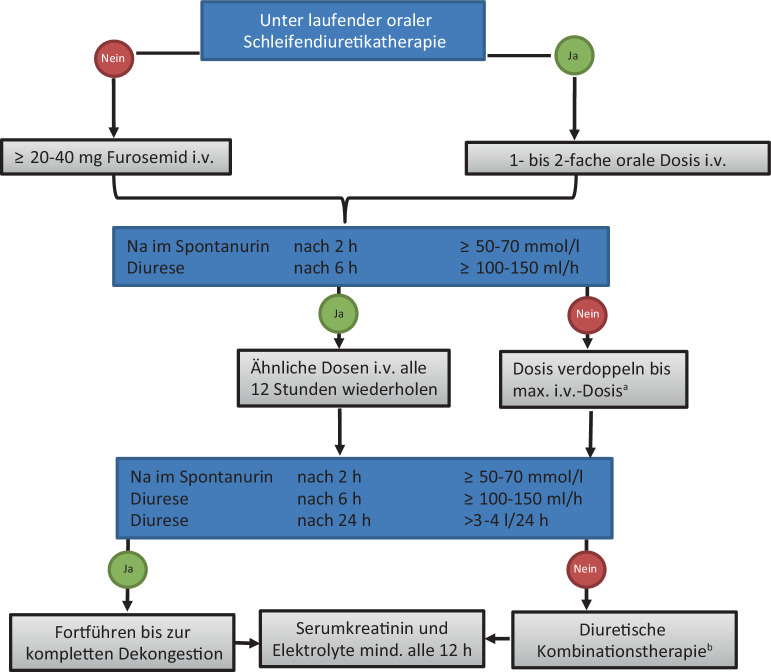
Diuretische Therapie bei dekompensierter Herzinsuffizienz. ^a^Maximale Furosemiddosis 400–600 mg/Tag, bei schwerer Niereninsuffizienz bis 1000 mg/Tag. ^b^Kombinationstherapie: Schleifendiuretikum + Thiazid/„thiazide-like diuretic“/Acetazolamid. (Adaptiert nach [21] und [20])

Schleifendiuretika

mit einer Klasse-I-Empfehlung zur Symptomkontrolle, Dekongestion und Erzielung einer optimalen Organperfusion [21]. Ein Schema zur praktischen Durchführung zeigt Abb. 5. Hier sei vor allem auf das frühe Monitoring der Natriurese hingewiesen, das im klinischen Alltag trotz einfacher Durchführbarkeit und geringer Kosten (noch) eher selten eine Umsetzung findet. Eine **adäquate Dekongestion**

Adäquate Dekongestion

ist mit einem **verbesserten Überleben**

Verbessertes Überleben

assoziiert [12]. Schleifendiuretika bewirken eine Natriurese und damit eine negative Wasser- und Salzbilanz und eine Dekongestion [22]. Die Kurve der Dosiswirkung von Schleifendiuretika verschiebt sich bei HI und CKD; daher ist meist eine höhere Dosis erforderlich, um die gleiche therapeutische Wirkung zu erzielen [23]. In einer Studie an 30 Patienten mit akuter kardialer Dekompensation und venöser Kongestion konnte gezeigt werden, dass durch eine leitliniengerechte Diuretikatherapie bei unverändertem Serumkreatinin die dopplersonographischen Parameter (IRVF, VII) sowie auch die Albuminurie signifikant verbessert werden [13].

In mehreren Studien wurden verschiedene **Dosierungsprotokolle**

Dosierungsprotokolle

bei akuter HI und ihre Auswirkungen auf die Nierenfunktion untersucht. Die bisher größte Studie ist die DOSE-AHF-Studie, in der 308 Patienten mit akuter HI randomisiert wurden und intravenös entweder eine niedrige (äquivalent zur oralen Erhaltungsdosis) oder eine hohe Dosis **Furosemid**

Furosemid

(2,5-fache orale Erhaltungsdosis) als Bolus oder als Dauerinfusion erhielten [24]. Die Ergebnisse der Studie zeigen keine signifikanten Unterschiede in den Symptomen oder Veränderungen der Nierenfunktion zwischen den beiden Gruppen. Allerdings ergaben sich bei den sekundären Endpunkten Atemnot, Nettoflüssigkeitsverlust und Gewichtsverlust nach 72 h in der Hochdosisgruppe bessere Werte als in der niedrig dosierten Gruppe. Es wurde jedoch kein signifikanter Unterschied in der mittleren Veränderung der Serumkreatininwerte zwischen den beiden Gruppen festgestellt. Schwerwiegende unerwünschte Ereignisse traten in der Hochdosisgruppe seltener auf als in der Niedrigdosisgruppe. Es wurden keine Dosierungen von Schleifendiuretika verwendet, die in der klinischen Praxis üblicherweise eingesetzt werden (mediane Dosis < 10 mg/h). Somit ist der Stellenwert der kontinuierlichen Infusion im Vergleich zur Bolusverabreichung unklar [11]. Die DOSE-AHF-Studie trug erheblich zur evidenzbasierten Medizin bei, jedoch zeigt die klinische Erfahrung, dass Schleifendiuretika allein häufig nicht ausreichend sind, um eine vollständige Dekongestion zu erzielen.

Vergleichende Untersuchungen zu Parametern der CN bei Bolus- oder kontinuierlicher Gabe liegen derzeit nicht vor.

#### Kombinationstherapien zur Steigerung der Natriumausscheidung/Entwässerung

Eine Ursache der **Diuretikaresistenz**

Diuretikaresistenz

(DR) ist die kompensatorische Erhöhung der **Natriumrückresorption**

Natriumrückresorption

in Bereichen des Nephrons, die nicht durch das (Mono‑)Diuretikum blockiert wird. Ein möglicher Ansatz zur Überwindung ist die Hinzunahme eines zweiten Diuretikums mit einem anderen Wirkmechanismus, auch bekannt als **sequenzielle Nephronblockade**

Sequenzielle Nephronblockade.

Die Anwendung einer Schleifendiurese wird in der Regel um die Einnahme eines **Thiaziddiuretikums**

Thiaziddiuretikum

als zweites Diuretikum ergänzt. Dieser Ansatz zielt auf einen **Synergieeffekt**

Synergieeffekt

ab, der durch die gleichzeitige Hemmung der Natriumrückresorption im distalen Tubulus erreicht wird. Wirksamkeit und Sicherheit dieser Kombination wurden in Studien nachgewiesen. Zu beachten ist jedoch, dass die Anwendung dieser Methode eine **sorgfältige Überwachung**

Sorgfältige Überwachung

erfordert, da das Risiko von Hypovolämie, Hypotonie, Hypokaliämie und einer Schädigung der Nierenfunktion erhöht ist [23]. Bei der Auswahl der Diuretika sollten auch deren Effekte auf den **Elektrolythaushalt**

Elektrolythaushalt

beachtet werden (Tab. 2). Ein Effekt einer diuretischen Kombinationstherapie auf die CN ist bis dato nicht belegt.

**Tab. 2:** Effekte diuretisch wirksamer Medikamente. (Nach [31])

	K^+^	Ca^2+^	Mg^2+^	Harnsäure	Na^+^	H^+^
*Schleifendiuretika*	↓	↓	↓	↑	↑	↓
*Thiazide*	↓	↑	↓	↑	↓	↓
*Carboanhydrasehemmer*	↓	↓	↓	←→	↓	↑
*Kaliumsparende Diuretika*	↑	↑^a^	↑	↑	↓↑^b^	↓↑
*SGLT-2-Inhibitoren*	←→/↑	←→	↑	↓	↑	←→
*Vaptane*	←→/↑	←→	←→	←→	↑	←→
*Osmotische Diuretika*	↓↑^c^	↓	↓	↓	↓↑	↑

^a^Spironolacton hat kaum Auswirkungen auf die Kalziumrückresorption

^b^ Amilorid und Triamteren sind mit einer Wasserausscheidung verbunden, während Spironolacton und Eplerenon mit einer Wasserretention verbunden sind

^c^Translokationshyponatriämie und Hyperkaliämie können während der Infusion auftreten, während Hypernatriämie und Hypokaliämie während der Diurese auftreten können

*SGLT‑2* Natrium-Glukose-Kotransporter 2

#### Sonderfall Diuretikaresistenz

Die Definition der DR wird kontrovers diskutiert. Gemeinhin gilt sie als die klinische Erscheinung einer nachlassenden diuretischen Wirkung, ohne das therapeutische Ziel der Ödemreduktion zu erreichen [23]. Andere Autoren definieren sie anhand laborchemischer Analysen dreier etablierter unabhängiger Kriterien:

- Fraktionierte Natriumausscheidung (FENa) < 0,2 %
- Na^+^ im Urin < 50 mmol/l
- Na^+^/K^+^-Quotient im Urin < 1,0

Unter den Bedingungen einer akuten kardialen Dekompensation war die DR durch ein Urinvolumen von < 100–150 ml/h in den ersten 6 h und einen Natriumgehalt im Urin von < 50–70 mmol/l in der zweiten Stunde nach Einnahme der Schleifendiuretika gekennzeichnet. Epidemiologische Studien zeigen, dass DR eng mit einer **schlechten Prognose**

Schlechte Prognose

und einem **erhöhten Sterberisiko**

Erhöhtes Sterberisiko

bei Patienten mit Nierenschäden oder Nephropathie verbunden ist. Die DR ist bei Patienten mit akuter kardiorenaler Dekompensation sehr häufig; die Inzidenz liegt bei 50–70 % [23]. Die Ursachen einer DR sind vielfältig. Ursachen in Zusammenhang mit einer CN sowie mögliche therapeutische Maßnahmen sind in Tab. 3 zusammengefasst. Eine wichtige Ursache in diesem Zusammenhang ist das sogenannte **Braking-Phänomen**

Braking-Phänomen.

Es beschreibt den Effekt, dass der Körper nach längerer Gabe von Diuretika eine abnehmende Reaktion auf diese Medikamente zeigt, vermittelt durch

- kompensatorische Mechanismen (RAAS-Aktivierung, erhöhte Sympathikusaktivität, Hypertrophie der Nierentubuli),
- Abnahme des Blutvolumens und
- Erhöhung der proximalen Natriumrückresorption.

Strategien zur Vermeidung und Behandlung umfassen

- diuretische Kombinationstherapien,
- passagere Pausen der Diuretikagaben,
- die Behandlung der RAAS-Aktivierung und/oder
- Anpassungen der Dosierung.

**Tab. 3:** Mechanismen der Diuretikaresistenz bei kongestiver Nephropathie und Maßnahmen zur Korrektur. (Modifiziert nach [31] und [32])

Ursache	Mechanismen	Korrekturmaßnahmen
**Patientenseitig**		
*Nichteinhaltung der empfohlenen Natrium- und/oder Flüssigkeitsbeschränkung*	Eine negative Salzbilanz wird nicht erreicht aufgrund einer „postdiuretischen Natriumretention“	Patientenschulung: Die 24 h-Natriurese sollte die Aufnahme übersteigen
*Medikamentöse Nonadhärenz*	Eine echte Resistenz tritt nur auf, wenn der Patient Diuretika in ausreichender Dosierung einnimmt, diese aber nicht wirksam sind	Patientenschulung
**Iatrogen**		
*Einnahme von NSAR*	Reduktion der Prostaglandinsynthese und renale Vasodilatation, interstitielle Nephritis	Medikamenteninteraktionen prüfen, NSAR absetzen
*Antihypertensiva*	Zu niedriger Blutdruck, reduzierter renaler Blutfluss, OAT-Interaktion	Dosis anpassen
*Probenecid*	Kompetition um OAT, veränderte Furosemid-Clearance	Medikamenteninteraktion prüfen
*Zu niedrige oder zu seltene Dosierung der Diuretika*	Mangelnde Überwachung der Ansprechparameter	Dosis erhöhen, bis die Ziele erreicht sind (Harnausscheidung, Natriurese, Dekongestion, klinische Verbesserung)
*Falsche Diagnose (z.* *B. venöses oder lymphatisches Ödem)*	Keine Berücksichtigung des klinischen Kontexts	Differenzialtherapie
**Begleiterkrankungen/-phänomene**		
*Braking-Phänomen*	Neurohormonelle Aktivierung, Hypertrophie der Tubuli	Diuretische Kombinationstherapien, passagere Pausen der Diuretika, Behandlung der RAAS-Aktivierung und/oder Anpassungen der Diuretikadosierung, Dialyse
*Metabolische Azidose und Urämie*	Beeinträchtigung der tubulären Resorption von Diuretika	Azidoseausgleich (Bicarbonat), Dialyse
*Akute/chronische Nierenschädigung*	Verminderte Nierendurchblutung	Wasserhaushalt überprüfen (ggf. Diuretika anpassen, Volumengabe), Dialyse
*Niedriger renaler Blutfluss*	Natriumretention durch Einschränkung der Filtration, Erhöhung der Natriumrückresorption und Verringerung der Abgabe von Diuretika im proximalen Tubulus	Vermeidung von Hypotonie, Bewertung des renalen Blutflusses mit Dopplersonographie
*Nephron-Remodeling*	Erhöhung der Natriumresorptionskapazität, Aktivierung des NaCl-Kanals, erhöhter transepithelialer Natriumtransport	Sequenzielle Nephronblockade
**Veränderte Pharmakokinetik**		
*Gleichzeitige Einnahme der Diuretika mit dem Essen*	Verzögerte Resorption, verminderte Spitzenkonzentration	Diuretika nüchtern einnehmen
*Schlechte Resorption*	Intestinales Ödem oder reduzierter intestinaler Blutfluss	Intravenöse Gabe
*Gestörte tubuläre Sekretion*	Medikamenteninteraktionen mit dem OAT	Interaktionen checken

*NSAR* nichtsteroidale Antirheumatika, *OAT* „organic anion transporter“, *RAAS* Renin-Angiotensin-Aldosteron-System

#### Nierenersatztherapie und Ultrafiltration

In einigen Fällen kann als **Rescue-Therapie**

Rescue-Therapie

eine Nierenersatztherapie oder Ultrafiltration in Betracht gezogen werden. Die Entscheidung ist komplex und erfolgt oft interdisziplinär. In diversen Studien wurde nachgewiesen, dass derartige Verfahren dazu beitragen können, Stauungen zu reduzieren, das neurohormonelle Gleichgewicht wiederherzustellen und die Lebensqualität der Patientinnen und Patienten zu verbessern. Allerdings erfordert ihre Anwendung eine sorgfältige Abwägung der potenziellen Vorteile und Risiken.

Eine Metaanalyse zur Effektivität einer Ultrafiltration bei akuter kardialer Dekompensation zeigt einen signifikanten Vorteil der Ultrafiltration im Vergleich zur konservativen Diuretikatherapie im Hinblick auf eine Verschlechterung der Herzfunktion, die Entwässerung, den erreichten Gewichtsverlust und die Rehospitalisierungsrate wegen akuter kardialer Dekompensation. Keine Unterschiede ergaben sich in den Endpunkten Nierenfunktionsverschlechterung und Gesamtsterblichkeit [25]. Daten zu Parametern der CN liegen nicht vor.

### Spezifische Interventionen

#### Inhibitoren des Renin-Angiotensin-Aldosteron-Systems

Die RAAS-Hemmung stellt eine etablierte Therapie für Patienten mit HI mit **reduzierter Pumpfunktion**

Reduzierte Pumpfunktion

(„heart failure with reduced ejection fraction“ [HFrEF]) dar und hat eine Klasse-I-Empfehlung. Zudem wurde in Studien nachgewiesen, dass eine RAAS-Hemmung das Fortschreiten einer CKD verlangsamt. Allerdings existieren nur wenige Studien, die sich mit den langfristigen nierenbezogenen Ergebnissen einer RAAS-Hemmung bei HI befassen, und keine Studien, die einen direkten Effekt auf Messgrößen der CN belegen [11].

#### Natrium-Glukose-Kotransporter-2-Inhibitoren

Ungeachtet der zahlreichen Daten zu **nephroprotektiven Effekten**

Nephroprotektive Effekte

von Natrium-Glukose-Kotransporter-2(SGLT-2)-Inhibitoren bei Patienten mit chronischer Niereninsuffizienz oder HI und/oder Diabetes mellitus [26] gibt es drei bemerkenswerte Studien zu Auswirkungen dieser Substanzklasse auf die CN. In einer prospektiven Beobachtungsstudie wurden Patienten mit chronischer Nierenerkrankung mit oder ohne Diabetes mellitus Typ 2 und/oder HI mit reduzierter oder erhaltener Ejektionsfraktion eingeschlossen, die eine Indikation für eine Standardtherapie mit SGLT-2-Inhibitoren aufwiesen. Nach einer 6‑monatigen Therapie mit SGLT-2-Inhibitoren wurde ein signifikanter Rückgang des mittleren VII der rechten Interlobarvenen und eine signifikante Verbesserung der IRVF-Muster beobachtet [27]. Eine weitere Studie verglich die Effekte einer frühen gegenüber einer verzögerten Initiierung einer SGLT-2-Inhibitor-Therapie (1,7 ± 0,4 vs. 6,9 ± 7,8 Tage nach stationäre Aufnahme) bei akuter kardialer Dekompensation. Durch die frühere Gabe konnte die Diurese wie auch die Länge des stationären Aufenthalts signifikant verbessert werden [28]. In einem Tiermodell mit venöser Kongestion führte eine SGLT-2-Inhibitor-Therapie zu einer Abnahme des renalen Ödems und zu einem Rückgang der interstitiellen Nierenfibrose, der Entzündung sowie der mitochondrialen Dysfunktion, insbesondere in der Nierenrinde [29].

#### Acetazolamid

Jüngste Studien, einschließlich der ADVOR-Studie, belegen, dass Acetazolamid in Kombination mit Schleifendiuretika zu einer schnelleren und umfassenderen **klinischen Dekongestion**

Klinische Dekongestion

bei akut dekompensierter HI führt, ohne jedoch das renale Outcome zu verbessern [30]. Diese Ergebnisse sind besonders relevant für Patienten mit DR und legen nahe, dass die Gabe von Acetazolamid als Zusatztherapie sinnvoll ist, wobei weitere Untersuchungen zur Sicherheit und zum potenziellen synergetischen Nutzen in der Behandlung von Patientinnen und Patienten mit HI erforderlich sind. Insbesondere fehlen Daten zur gleichzeitigen Anwendung von Acetazolamid und SGLT-2-Inhibitoren. Daten zur CN liegen gleichfalls nicht vor.

##### Merke.

Schleifendiuretika und SGLT-2-Inhibitoren führen nachweislich zu einer Dekongestion. Bei DR sollten differenzialdiagnostische und -therapeutische Erwägungen erfolgen.

## Häufige Probleme bei CN

Im Zusammenhang mit einer CN treten drei Probleme gehäuft auf:

- „Worsening and pseudo-worsening renal function“
- Hyperkaliämie
- Hyponatriämie

### „Worsening and pseudo-worsening renal function“

Man unterscheidet eine Verschlechterung der Nierenfunktion („worsening renal function“ [**WRF**

WRF])

von einer Pseudoverschlechterung („pseudo-worsening renal function“ [**Pseudo-WRF**

Pseudo-WRF]).

Beiden gemein ist ein **Serumkreatininanstieg**

Serumkreatininanstieg

im Rahmen einer HI bzw. unter deren Therapie. Wie hoch dabei der Anstieg des Serumkreatinins sein muss, ist noch Gegenstand von Debatten. In Anlehnung an die Definition der akuten Nierenschädigung wurde in den meisten Berichten ein Anstieg von > 0,3 mg/dl (26,5 µmol/l) genutzt. Diese Definition berücksichtigt jedoch nicht die exponentielle Beziehung zwischen Serumkreatinin und eGFR, sodass je nach absoluter Höhe des Ausgangskreatinins entweder kleine oder große Veränderungen der tatsächlichen Nierenfunktion mit einer derartigen Veränderung einhergehen können [31]. Eine WRF ist mit **schlechtem Outcome**

Schlechtes Outcome

assoziiert, aber nur wenn sich dazu parallel der klinische Zustand des Patienten verschlechtert.

Verbessert sich der klinische Zustand im Rahmen der Rekompensationstherapie bei steigendem Serumkreatinin, bezeichnet man das als Pseudo-WRF. Dies ist prognostisch als eher günstig anzusehen. In mehreren retrospektiven Analysen großer randomisierter, kontrollierter **RAAS-Hemmer-Studien**

RAAS-Hemmer-Studien

wurde gezeigt, dass die positive Wirkung entsprechender Therapien erhalten bleibt, wenn die Pseudo-WRF bei der Initiierung dieser Therapien auftritt, insbesondere bei ACE-Hemmern, Angiotensinrezeptorblockern und Mineralokortikoidrezeptorantagonisten [31]. Auch für eine **diuretische Dekongestionstherapie**

Diuretische Dekongestionstherapie

konnte gezeigt werden, dass das Überleben sich trotz steigender Serumkreatininwerte verbessert, sofern eine Hämokonzentration erreicht wird [33]. Kleine bis mäßige Abnahmen der GFR, wie man sie häufig während einer aggressiven Diurese beobachtet, sind am ehesten kein Ausdruck einer tubulären Schädigung der Niere, sondern wahrscheinlich „klinisch gutartige“ Ereignisse bedingt durch eine veränderte Filtration [34]. Analysen von **Biomarkern**

Biomarker

einer tubulären Schädigung könnten in Zukunft zur weiteren Aufklärung beitragen.

Eine erweiterte **differenzialdiagnostische Abklärung**

**Abb. 6:**
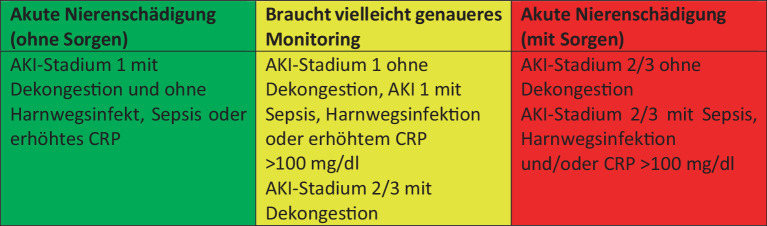
Ampelsystem für das Management einer De-novo-AKI unter Therapie bei Patienten mit akut dekompensierter Herzinsuffizienz. *AKI* „acute kidney injury“ (akute Nierenschädigung), *CRP* C‑reaktives Protein. (Nach [12])

Differenzialdiagnostische Abklärung

einer Serumkreatininveränderung sollte nach dem Schema in Abb. 6 erfolgen [12]). Eine gute Übersicht zum differenzialdiagnostischen Vorgehen findet man bei Damman et al. [31] und Mullens et al. [20].

### Hyperkaliämie

Hyperkaliämien sind bei CN häufig. Es ist entscheidend, ob es sich um eine akute (bedrohliche) oder eine chronische Hyperkaliämie handelt. Eine HI-spezifische Medikation sollte so lange wie möglich beibehalten werden [35]. Die Behandlung der chronischen Hyperkaliämie beruht im Wesentlichen auf vier Säulen:

- Diätetische Maßnahmen
- Normalisierung des Bicarbonatspiegels (Verschreibung von Natriumbicarbonat 1–3 g/Tag, um einen Serumspiegel von mehr als 22 mmol/l zu erreichen)
- Steigerung der renalen Ausscheidung mit Schleifendiuretika oder Thiaziden (bei Patienten mit Volumenüberladung)
- Verbesserung der Kaliumausscheidung über den Magen-Darm-Trakt mit kaliumbindenden Medikamenten, z. B. Natrium-Zirkonium-Zyklosilikat oder Patiromer [36]

### Hyponatriämie

Eine Hyponatriämie (< 135 mmol/l) tritt bei bis zu 30 % der hospitalisierten Patienten mit HI auf, häufig in Verbindung mit DR. Sie ist mit einem erhöhten Risiko für **Komplikationen**

Komplikationen

wie Stürze, Frakturen und kognitive Beeinträchtigungen sowie mit längeren Krankenhausaufenthalten und höherer Gesamtmortalität verbunden. Bei CN zeigt sich häufig eine **hypervolämische Verdünnungshyponatriämie**

Hypervolämische Verdünnungshyponatriämie,

die im Verlauf durch die diuretische Therapie in eine **hypovolämische Depletionshyponatriämie**

Hypovolämische Depletionshyponatriämie

übergehen kann. Jede dieser Varianten erfordert unterschiedliche therapeutische Ansätze. Ein frühzeitiger Beginn und eine Dosissteigerung von leitliniengerechten Wirkstoffen wie ACE-Hemmern und Betablockern können die Hyponatriämie bei Patienten mit HI verbessern, während Mineralokortikoidantagonisten sie möglicherweise verschlechtern [35].

#### Cave.

Ein steigendes Serumkreatinin im Rahmen einer CN bzw. unter deren Therapie bedarf einer differenzialdiagnostischen Beurteilung. Bei gleichzeitiger klinischer Besserung ist dies am ehesten als Pseudo-WRF und damit prognostisch als günstig zu werten. Andere Differenzialdiagnosen dürfen jedoch nicht außer Acht gelassen werden, insbesondere bei ungünstigem klinischem Verlauf (Abb. 6).

## Fallbeispiel (Fortsetzung)

Die Therapie wird zunächst auf intravenöse Schleifendiuretika umgestellt. Ramipril bei kritischer Hyperkaliämie und Metformin bei akuter Nierenschädigung werden zunächst pausiert und eine Therapie mit Natrium-Zirkonium-Zyklosilikat, Natriumbicarbonat und Empagliflozin begonnen. Darunter wird eine Gewichtsreduktion von 6 kg in 6 Tagen erzielt. Es kommt zu einer klinischen Besserung der Symptomatik (NYHA-Stadium II), die eGFR steigt auf 42 ml/min pro 1,73 m^2^, der Kaliumspiegel normalisiert sich, der pH-Wert ist ausgeglichen. Eine Therapie mit Sacubitril/Valsartan und einem niedrig dosierten Mineralokortikoidantagonisten wird initiiert. Vor Entlassung wird die diuretische Therapie auf die orale Gabe von Torasemid umgestellt und die Metformingabe wieder begonnen. Die Ergebnisse der IRD vor Entlassung zeigen einen kontinuierlichen Blutfluss. Der Patient wird an die kardiorenale Spezialsprechstunde angebunden.

## Renokardiales Syndrom

Das renokardiale Syndrom beschreibt die **wechselseitige Beeinflussung**

Wechselseitige Beeinflussung

zwischen akuter oder chronischer Niereninsuffizienz und Herzfunktion sowie die daraus resultierenden, sekundären Beeinträchtigungen der Herz- bzw. Nierenfunktion. Kardiovaskuläre Erkrankungen sind bei CKD-Patienten mit einer Prävalenz von etwa 67 % deutlich häufiger als bei Individuen ohne CKD, wobei die HI über alle CKD-Stadien mit zunehmender Prävalenz bei nachlassender GFR ungefähr 4‑mal so häufig vorkommt [37]. CKD-Patienten mit kardiovaskulären Erkrankungen haben ein erhöhtes Risiko für Hospitalisierungen, Intensivpflichtigkeit und kardiovaskulären Tod [38]. Die pathophysiologischen Mechanismen beruhen auf

- einer Aktivierung des RAAS und des sympathischen Nervensystems,
- einer vermehrten Flüssigkeits- und Natriumretention,
- einem erhöhten Blutdruck,
- einer (renalen) Anämie,
- urämiebedingten Mechanismen,
- einer renalen Azidose,
- oxidativem Stress und Inflammation sowie
- Störungen des Kalzium-Phosphat-Stoffwechsels.

Die Behandlung des renokardialen Syndroms folgt einem **multidisziplinären Ansatz**

Multidisziplinärer Ansatz.

Die wesentlichen Inhalte werden im weiteren Text beschrieben.

### Kausale Behandlung der zugrunde liegenden Nierenerkrankung

Es ist von entscheidender Bedeutung, sofern möglich eine **ätiologische Diagnose**

Ätiologische Diagnose

der CKD zu stellen, denn dies ermöglicht eine gezielte Therapie, beispielsweise eine Immunsuppression. Dafür ist neben **serologischen Analysen**

Serologische Analysen

gegebenenfalls auch die **histologische Untersuchung**

Histologische Untersuchung

der Niere erforderlich. Die Indikation zur Nierenbiopsie muss jedoch im Kontext der vorliegenden Befunde abgewogen werden [39]. Unabhängig von histologischen Analysen konnte für SGLT-2-Inhibitoren bei CKD [40, 41] wie auch für den nichtsteroidalen Mineralokortikoidantagonisten Finerenon bei diabetischer Nephropathie [42] eine signifikante Reduktion der Hospitalisierungsrate wegen dekompensierter HI nachgewiesen werden.

### Inhibition des Renin-Angiotensin-Aldosteron-Systems und Flüssigkeitsmanagement

Diese Therapieansätze sind weiter oben beschrieben.

### Blutdruckkontrolle

Eine gute Blutdruckkontrolle dient unstrittig der Hemmung einer **CKD-Progression**

CKD-Progression,

aber auch der Verhinderung einer akuten kardialen Dekompensation [43, 44]. Noch nicht vollständig geklärt sind jedoch die Zielwerte bzw. das genaue Vorgehen. Während eine **intensive Blutdrucksenkung**

Intensive Blutdrucksenkung

in der Interventionsphase der SPRINT-Studie mit einer signifikant stärkeren Reduktion der eGFR (−0,96 vs. −0,67 ml/min pro Jahr, *p* < 0,001) und einem erhöhten Risiko für nierenbedingte Ereignisse assoziiert war, war der postinterventionelle „GFR slope“ (Veränderung der GFR pro Zeiteinheit) in der Interventionsgruppe tendenziell niedriger (−0,85 vs. −1,02 ml/min pro Jahr, *p* = 0,28; [45]). In der Studie konnte eine signifikante Interaktion zwischen der Ausgangs-eGFR und den Behandlungsgruppen in Bezug auf die Prävention einer **kardialen Dekompensation**

Kardiale Dekompensation

beobachtet werden (Interaktion *p* = 0,012). Die Reduktion des Risikos einer kardialen Dekompensation zeigte sich bis zu einer eGFR von 75 ml/min. Eine intensive Blutdruckbehandlung hatte keinen präventiven Effekt (Hazard Ratio 1,03; 95 %-Konfidenzintervall 0,82–1,52), wenn die Baseline-eGFR ≤ 75 ml/min betrug [46].

### Anämiebehandlung

Eine **Eisensubstitution**

Eisensubstitution

bei Eisenmangel ist sowohl für die Indikation HFrEF bzw. „heart failure with mildly reduced ejection fraction“ (HFmrEF; [21]) zur Verbesserung der Belastbarkeit (Empfehlungsgrad 1A) und Reduktion der Hospitalisierung wegen dekompensierter HI (Empfehlungsgrad 2A) als auch bei CKD (Empfehlungsgrad 2D) empfohlen [58]. Eine Therapie mit **erythropoesestimulierenden Agenzien**

Erythropoesestimulierende Agenzien

(ESA) sollte bei nicht dialysepflichtigen Patienten mit CKD in Abhängigkeit von den Symptomen und Begleiterkrankungen initiiert werden (Empfehlungsgrad 2D; [58]). Eine Cochrane-Metaanalyse von 11 Studien bei anämischen Patienten mit HI zeigte, dass eine Verabreichung von ESA zu einer Verbesserung der Belastungstoleranz und zur Abnahme der HI-bedingten Krankenhausaufenthalte führte [47]. Die ASCEND-ND-Studie zeigte bei nicht dialysepflichtigen Patienten mit CKD eine signifikant höhere Inzidenz im Hinblick auf durch akut dekompensierte HI bedingte Hospitalisierungen unter einer Therapie mit dem Hypoxia-inducible-factor(HIF)-Stabilisator **Daprodustat**

Daprodustat

im Vergleich zu konventionellen ESA [48].

### Urämie

Mit Ausnahme der Behandlung des Auslösers (beispielsweise einer Exsikkose) und der Dialyse gibt es keine supportive Therapie, die akut zu einer Verbesserung des urämischen Milieus führt.

### Behandlung der metabolischen Azidose

Der Einsatz von **Bicarbonat**

Bicarbonat

zum Ausgleich einer metabolischen Azidose führte bei Patienten mit CKD zu einer signifikanten Abnahme von „major adverse cardiovascular events“ [MACE]), der HI-Inzidenz, der Hospitalisierungsrate wegen Lungenödem wie auch der Mortalität [49, 50]. Die Leitlinien von Kidney Disease: Improving Global Outcomes (KDIGO) empfehlen deshalb, eine **pharmakologische Behandlung**

Pharmakologische Behandlung

mit oder ohne **diätetische Maßnahmen**

Diätetische Maßnahmen

bei einem Serumbicarbonatspiegel < 18 mmol/l zu erwägen [51].

### Management der Störungen des Kalzium-Phosphat-Stoffwechsels

Der mit einer CKD einhergehenden **Hyperphosphatämie**

Hyperphosphatämie

und dem einhergehenden **Hyperparathyreoidismus**

Hyperparathyreoidismus

wird eine Schlüsselrolle in Bezug auf Klappen‑/Gefäßverkalkung und -steifigkeit sowie die damit verbundenen kardiovaskulären Ereignisse zugeschrieben. Es gibt einen Zusammenhang zwischen Phosphatspiegel und Auftreten kardialer Ereignisse [52]. In der multizentrischen randomisierten, kontrollierten Studie IMPROVE-CKD werden derzeit die Effekte des Phosphatsenkers **Lanthancarbonat**

Lanthancarbonat

auf die Pulswellengeschwindigkeit und aortal-abdominelle Kalzifikation bei 278 Patienten mit den CKD-Stadien 3b–4 untersucht [53]. Prospektive, randomisierte Daten, in denen protektive Effekte einer Phosphatsenkung und/oder Absenkung des Parathormons auf das kardiovaskuläre Outcome beschrieben werden, liegen derzeit nicht vor [54, 55].

#### Merke.

Die Behandlung des renokardialen Syndroms beinhaltet

- eine kausale Behandlung der zugrunde liegenden Nierenerkrankung,
- Blutdruckkontrolle,
- Flüssigkeitsmanagement,
- Anämiebehandlung,
- die Behandlung der metabolischen Azidose und
- ein Management der Störungen des Kalzium-Phosphat-Stoffwechsels.

## Unbeantwortete Fragen

Obwohl das Phänomen der CN seit mehr als einem Jahrhundert bekannt ist [56], gibt es bis zum heutigen Tag keine allgemein akzeptierten Diagnosekriterien. In Ermangelung dessen sind die Prävalenzen der CN bei den verschiedenen auslösenden Erkrankungen nicht genau bekannt. In der klinischen Praxis erfolgt die Dekongestion in der Regel durch Gabe von Diuretika. Die Steuerung der Therapie erfolgt dabei anhand klinischer Parameter. Ob eine Steuerung anhand noch zu definierender Kongestionsparameter im Hinblick auf Morbidität oder Mortalität von Vorteil ist, kann derzeit nicht beantwortet werden.

## Fazit für die Praxis

- Bei Vorliegen prädisponierender Erkrankungen und einem relevanten Abfall der errechneten glomerulären Filtrationsrate (eGFR) sollte differenzialdiagnostisch auf Zeichen der venösen Kongestion geachtet werden.
- Derzeit ist die (Doppler‑)Sonographie die Methode, mit der eine kongestive Nephropathie (CN) am besten erfasst werden kann.
- Sonographisch ist auf den Durchmesser der V. cava inferior und auf deren Atemmodulation zu achten.
- Dopplersonographisch können das intrarenale venöse Flussprofil, der Venous Impedance Index und der Renal Venous Stasis Index zur Beurteilung der Kongestion herangezogen werden.
- Diuretika und Natrium-Glukose-Kotransporter-2(SGLT-2)-Inhibitoren verbessern die dopplersonographischen Parameter der venösen Kongestion.

## CME-Fragebogen

Welche der folgenden Erkrankungen ist – sofern sie isoliert auftritt – am häufigsten mit einer kongestiven Nephropathie assoziiert?

Aortenklappeninsuffizienz

Mitralklappenstenose

Trikuspidalklappeninsuffizienz

Pulmonalklappeninsuffizienz

Aortenklappenstenose

Welche Aussage zur Pathogenese der kongestiven Nephropathie ist richtig?

Die kongestive Nephropathie wird durch eine arterielle Minderperfusion der Nieren ausgelöst.

Die Aktivierung des Renin-Angiotensin-Aldosteron-Systems (RAAS) ist bei kongestiver Nephropathie nicht beteiligt.

Die kongestive Nephropathie entsteht durch einen selektiven Verlust der glomerulären Filtrationsrate ohne Einfluss auf den interstitiellen Druck.

Die Pathogenese der kongestiven Nephropathie wird durch eine primäre Schädigung der Tubuluszellen bestimmt.

Erhöhter zentralvenöser Druck führt zu einer Einschränkung der Nierenfunktion durch gestörten venösen Abfluss und erhöhten Kapillardruck.

Welches Flussmuster in den Nierenvenen entspricht am ehesten der schwersten Form der kongestiven Nephropathie?

Kontinuierlich

Pulsatil

Monophasisch

Biphasisch

Triphasisch

Welche Aussage zu den Auswirkungen diuretisch wirksamer Medikamente trifft zu?

Schleifendiuretika erhöhen den Mg^2+^-Spiegel.

Thiaziddiuretika senken die Harnsäure.

Vaptane senken das Serumnatrium.

Carboanhydrasehemmer erhöhen den Ca^2+^-Spiegel.

Spironolacton kann eine Hyperkaliämie erzeugen.

Welche dopplersonographische Messgröße spricht am ehesten für das Vorliegen einer kongestiven Nephropathie?

Widerstandsindex RI = 0,65

Index der venösen Impedanz (Venous Impedance Index [VII]) = 0

Index der renalvenösen Stase (Renal Venous Stasis Index [RVSI]) = 0,1

Maximale systolische Flussgeschwindigkeit in der Interlobararterie V_s_ = 35 cm/s

Systolische Flussgeschwindigkeit in der Interlobarvene V_s_ = 11 cm/s

Welche der nachfolgenden Beschreibungen erklärt am ehesten eine „pseudo-worsening renal function“?

Kreatininanstieg um 1,0 mg/dl im Rahmen eines kardiogenen Schocks

Kreatininanstieg von 0,6 auf 1,0 mg/dl bei kardialer Dekompensation New-York-Heart-Association(NYHA)-Stadium III und unbehandeltem Harnwegsinfekt

Kreatininanstieg von 1,2 auf 1,8 mg/dl bei bekannter Herzinsuffizienz mit reduzierter Ejektionsfraktion und Nierenstau III°

Kreatininanstieg von 0,9 auf 1,8 mg/dl bei einem Patienten mit Herzinsuffizienz mit erhaltener Ejektionsfraktion (HFpEF) drei Tage nach Durchführung einer Koronarangiographie

Kreatininanstieg von 1,5 auf 2,1 mg/dl bei einem Patienten mit kardialer Dekompensation New-York-Heart-Association(NYHA)-Stadium IV und suffizienter diuretischer Therapie (Gewichtsabnahme um 5,2 kg in 7 Tagen, „downgrading“ auf NYHA-Stadium II)

Welche Aussage zur „worsening renal function“ (WRF) im Rahmen einer kongestiven Nephropathie ist richtig?

WRF ist ein irreversibler Zustand, der zwangsläufig zu einer Dialyse führt.

WRF tritt bei Patienten mit schwerer Herzinsuffizienz im Endstadium auf.

Die wichtigste Ursache für WRF bei kongestiver Nephropathie ist eine Unterperfusion der Nieren durch niedrigen Blutdruck.

Die Prognose einer WRF ist schlechter als bei einer Pseudo-WRF.

Eine Verschlechterung der Nierenfunktion im Rahmen einer kongestiven Nephropathie bedeutet, dass die Diuretika vollständig abgesetzt werden müssen.

Welche der nachfolgenden Medikamente/Substanzklassen führt nachweislich zu einem Rückgang des Kongestionsgrads in der intrarenalen Dopplersonographie?

Angiotensin-converting-enzyme(ACE)-Hemmer

Angiotensinrezeptor-Neprilysin-Inhibitoren (ARNI)

Betablocker

Natrium-Glukose-Kotransporter-2(SGLT-2)-Inhibitoren

Vericiguat

Welche der nachfolgenden Definitionen beschreibt eine Diuretikaresistenz am ehesten?

Diuretikaresistenz bedeutet eine unzureichende Entwässerung des Körpers auf Diuretika trotz angemessener Dosierung und Verabreichung.

Diuretikaresistenz ist eine Allergie gegen Diuretika.

Diuretikaresistenz bedeutet einen Anstieg des Serumkreatinins auf mehr als das Dreifache des Ausgangswerts auf die Gabe eines Schleifendiuretikums.

Diuretikaresistenz liegt vor bei Eintritt einer Hyponatriämie bei Überdosierung von Diuretika.

Diuretikaresistenz liegt vor bei Diuresen < 4000 ml/24 h nach Gabe von 80 mg Furosemid i.v.

Welche Aussage beschreibt die wesentlichen Säulen der Behandlung einer chronischen Hyperkaliämie bei kongestiver Nephropathie korrekt?

Die Behandlung erfolgt durch den sofortigen Abbruch aller herzinsuffizienzspezifischen Medikamente.

Kaliumbindende Medikamente wie Natrium-Zirkonium-Zyklosilikat oder Patiromer spielen eine Rolle bei der Verbesserung der Kaliumausscheidung über den Magen-Darm-Trakt.

Eine Ernährung mit erhöhtem Kaliumgehalt wird empfohlen, um die renale Ausscheidung zu fördern.

Die Normalisierung des Bicarbonatspiegels ist nicht relevant für die Behandlung der Hyperkaliämie.

Schleifendiuretika und Thiazide sind bei der Behandlung von Hyperkaliämie wirkungslos, da sie den Kaliumspiegel nicht beeinflussen.
